# UBL3 Interaction with α-Synuclein Is Downregulated by Silencing MGST3

**DOI:** 10.3390/biomedicines11092491

**Published:** 2023-09-08

**Authors:** Jing Yan, Hengsen Zhang, Yuna Tomochika, Bin Chen, Yashuang Ping, Md. Shoriful Islam, Shuhei Aramaki, Tomohito Sato, Yu Nagashima, Tomohiko Nakamura, Tomoaki Kahyo, Daita Kaneda, Kenji Ogawa, Minoru Yoshida, Mitsutoshi Setou

**Affiliations:** 1 Department of Cellular and Molecular Anatomy, Hamamatsu University School of Medicine, 1-20-1 Handayama, Higashi-ku, Hamamatsu 431-3192, Japan; 2 Department of Radiation Oncology, Hamamatsu University School of Medicine, 1-20-1 Handayama, Higashi-ku, Hamamatsu 431-3192, Japan; 3 International Mass Imaging Center, Hamamatsu University School of Medicine, 1-20-1 Handayama, Higashi-ku, Hamamatsu 431-3192, Japan; 4 Institute for Medical Photonics Research, Preeminent Medical Photonics Education and Research Center, Hamamatsu University School of Medicine, 1-20-1 Handayama, Higashi-ku, Hamamatsu 431-3192, Japan; 5 Department of Neurology, Hamamatsu University School of Medicine, 1-20-1 Handayama, Higashi-ku, Hamamatsu 431-3192, Japan; 6 Choju Medical Institute, Fukushimura Hospital, Yamanaka-19-14 Noyoricho, Toyohashi 441-8124, Japan; 7 Laboratory of Veterinary Epizootiology, College of Bioresource Sciences, Nihon University, Fujisawa 252-0880, Japan; 8 Department of Biotechnology, Graduate School of Agricultural and Life Sciences, The University of Tokyo, Tokyo 113-8657, Japan; 9 Collaborative Research Institute for Innovative Microbiology, The University of Tokyo, Tokyo 113-8657, Japan; 10 RIKEN Center for Sustainable Resource Science, Wako 351-0198, Japan; 11 Department of Systems Molecular Anatomy, Institute for Medical Photonics Research, Preeminent Medical Photonics, Education & Research Center, 1-20-1 Handayama, Higashi-ku, Hamamatsu 431-3192, Japan

**Keywords:** ubiquitin-like 3, α-synuclein, microsomal glutathione s-transferase 3, oxidative stress, hydrogen peroxide

## Abstract

Ubiquitin-like 3 (UBL3) is a membrane-anchored protein that plays a crucial role in sorting proteins into small extracellular vesicles. Aggregations of alpha-synuclein (α-syn) are associated with the pathology of neurodegenerative diseases such as Parkinson’s disease. Recently, the interaction between UBL3 and α-syn was discovered, with potential implications in clearing excess α-syn from neurons and its role in disease spread. However, the regulator that can mediate the interaction between UBL3 and α-syn remains unclear. In this study, using the split gaussian luciferase complementation assay and RNA interference technology, we identified that QSOX2, HTATIP2, UBE3C, MGST3, NSF, HECTD1, SAE1, and ATG3 were involved in downregulating the interaction between UBL3 and α-syn. Notably, silencing MGST3 had the most significant impact. Immunocytochemistry staining confirmed the impact of MGST3 silencing on the co-localization of UBL3 and α-syn in cells. MGST3 is a part of the antioxidant system, and silencing MGST3 is believed to contribute to oxidative stress. We induced oxidative stress with hydrogen peroxide, observing its effect on the UBL3-α-syn interaction, and showing that 800 µM of H_2_O_2_ downregulated this interaction. In conclusion, silencing MGST3 downregulates the interaction between UBL3 and α-syn.

## 1. Introduction

Ubiquitin-like 3 (UBL3), also known as membrane-anchored ubiquitin-fold protein (MUB), is a highly conserved ubiquitin-like protein [1]. In 2018, it was discovered that UBL3 can serve as a post-translational modifier of proteins, tagging them for sorting into small extracellular vesicles (sEVs) [2]. sEVs are secreted by various cells and have a diameter of 30–150 nm [3]. They play crucial roles in mediating cell-to-cell communication in both physiology and pathology. For instance, sEVs that transport proteins between cells have been implicated in the development of neurodegenerative diseases [4].

Alpha-synuclein (α-syn) is encoded by the SNCA gene, which consists of 140 amino acids and weighs 14.5 kDa. It is highly expressed in the adult central nervous system, especially in presynaptic terminals [5]. Abnormal aggregations of α-syn are a major feature of Lewy bodies, which are found in neurons of patients with neurodegenerative diseases such as sporadic Parkinson’s disease (PD) and Dementia with Lewy bodies (DLB) [6,7]. Research has shown that the interactions of α-syn with specific proteins can influence its aggregation and toxicity. For example, ꞵ-amyloid peptides can interact with α-syn and promote the intraneuronal accumulation of α-syn [8] whereas heat shock protein 70 (Hsp70) can interact with α-syn and aid in its clearance, thereby reducing its aggregation [9]. The modulation of these interactors is emerging as a promising therapeutic strategy for neurodegenerative diseases.

Recently, the interaction of UBL3 with α-syn has been revealed using split gaussian luciferase complementation assay and immunoprecipitation [10]. This interaction is thought to be related to the clearance of excess α-syn from neurons, or the propagation of α-syn pathology in the brain associated with neurodegenerative diseases. However, the regulators involved in regulating the interaction of UBL3 with α-syn are currently unknown.

In this study, we used split gaussian luciferase complementation assay and RNA interference (RNAi) technology to discover that silencing MGST3 downregulates the interaction of UBL3 with α-syn.

## 2. Materials and Methods

### 2.1. Plasmids and siRNA

The NGluc-UBL3, α-syn-CGluc, Flag-UBL3, MYC-α-syn, and Gluc plasmids were previously used in our laboratory [10]. The siRNAs used for silencing target candidate proteins purchased were Ambion Silencer Select (Life Technologies, Carlsbad, CA, USA) (Supplementary Table S1). Two siRNAs were available for each candidate protein. The complementary mRNAs position and the target exons of the two siRNAs in each candidate protein are different. One additional siRNA was provided as a negative control group to assure silencing efficiency and avoid siRNA toxicity.

### 2.2. Cell Culture and Transfection

Human embryonic kidney (HEK293) cells (RIKEN Cell Bank, Tsukuba, Japan) were cultured in Dulbecco’s Modified Eagle Medium (DMEM, GIBCO, 11965-092) supplemented with 10% fetal bovine serum (FBS) at 37 °C in a humidified incubator with 5% CO_2_.

The cells were seeded in 24-well plates. When the cell confluency reached 70–80%, transfection was performed using Lipofectamine 2000 (Thermo Fisher Scientific, Waltham, MA, USA) and Opti-MEM reduced serum medium (Thermo Fisher Scientific, Waltham, MA, USA). Specifically, 100 ng of NGluc-UBL3 plasmid, 100 ng of α-syn-CGluc plasmid, and 20 pmol of siRNA were transfected per well. The transfection was performed according to the manufacturer’s instruction for Lipofectamine 2000. 

### 2.3. Luciferase Assay

Cells were incubated for 72 h after transfection. We collected the cell culture medium and centrifuged it at 1200 rpm for 5 min to remove cell debris. Then, the supernatant was added to 17 μg/mL coelenterazine (Cosmo Bio, Kyodo, Japan) in Opti-MEM and luminescence was immediately measured using BioTek Synergy H1 microplate reader (Agilent, Santa Clara, CA, USA).

### 2.4. Western Blot

Cells were washed with ice-cold PBS and harvested by centrifugation at 2000× *g* for 5 min at 4 °C. Then, the cells were resuspended and lysed with 1% Triton lysate buffer (50 mM Tris-HCl [pH 7.4], 100 mM NaCl, and 1% [v/v] Triton X-100) for 30 min on ice. Cell lysate was centrifuged at 20,000× *g* for 15 min at 4 °C to remove cell debris and un-lysed cells. Quantification of protein concentration was performed using the Pierce BCA Protein Assay Kit (23227, Thermo Fisher Scientific, Waltham, MA, USA). For WB analysis, 10 μg of total protein was loaded after being treated with 2-mercaptoethanol (βME) sodium dodecyl sulfate (SDS) sample loading buffer (100 mM Tris-HCl [pH 6.8], 4% SDS, 20% glycerol, and 0.01% bromophenol blue) at 95 °C for 5 min, separated on SDS-PAGE gel and transferred to polyvinylidene fluoride (PVDF) membrane. The membrane was blocked with 5% skim milk for 1 h at room temperature and then incubated overnight at 4 °C with the anti-MGST3 antibody (Abcam, ab192254; 1:1000, Cambridge, UK) and anti-β-Actin antibody (Cell Signaling Technology, 8H10D10, 1:1000 dilution, Danvers, MA, USA). Subsequently it was incubated with horseradish peroxidase (HRP)-conjugated secondary antibody (Cell Signaling Technology, 1:5000 dilution) for 1 h at room temperature after washing. We detected the protein signal using the Enhanced Chemiluminescence Kit (32106, Thermo Fisher Scientific, Waltham, MA, USA) with the FUSION FX imaging system (Vilber Lourmat, Collégien, Seine-et-Marne, France).

### 2.5. Immunocytochemistry

HEK293 cells were co-transfected with Flag-UBL3, MYC-α-syn, and MGST3 siRNA (or negative-control siRNA) using Lipofectamine 2000. After 24 h, cells were fixed with methanol for 5 min; blocked with 1% BSA/PBS for 1 h; incubated with primary antibodies, anti-Flag antibodies (MERCK, F7425-.2 MG, 1:500 dilution, Rahway, NJ, USA) and anti-MYC antibodies (MBL, M1923, 1:500 dilution, Woods Hole, MA, USA) for 18 h at 4 °C, and secondary antibodies (Invitrogen, Alexa Fluor 488, Alexa Fluor 647, 1:500 dilution, Carlsbad, CA, USA) for 1 h; and mounted with VECTASHIELD Mounting Medium (Vector). Confocal images were acquired with a 63× objective lens on a confocal laser microscope (Leica TCS SP8). The colocalizations of UBL3 with α-syn were analyzed using ImageJ 2.0 software.

### 2.6. Oxidative Stress

We used hydrogen peroxide (H_2_O_2_) (FUJIFILM, Japan) to induce oxidative stress. After transfection, the cells were passaged into 96-well plates with 100 μL of DMEM (10% FBS) per well and incubated overnight. The H_2_O_2_ was diluted with pre-warmed DMEM (10% FBS). We replaced the culture medium in each well with culture medium containing different concentrations of H_2_O_2_. The final H_2_O_2_ concentrations were 0 µM, 100 µM, 200 µM, 400 µM, and 800 µM. Cells were incubated for a further 48 h, and the culture medium was collected for luciferase assay.

### 2.7. MTT Assay

The 3-(4,5-dimethylthiazol-2-yl)-2,5-diphenyl-2H-tetrazolium Bromide (MTT) cell growth kit (CT02, Millipore, MA, USA) was used for measuring cell viability. After collecting cell culture medium for luciferase assay, 100 μL pre-warmed DMEM (10% FBS) medium was added to each well with 10 μL of MTT reagent and incubated for 4 h. Then, we added 100 μL solubilization buffer, isopropanol with 0.04 N hydrogen chloride, and the absorbance value (OD) at 450 nm wavelength was measured using the microplate reader. Cell viability equal to (OD Intervention group—OD Blank group)/(OD Control group—OD Blank group). The blank group contained only medium without cells and the control group had medium and cells without intervention.

### 2.8. Statistical Analysis

Statistical analysis was performed using GraphPad Prism 8.0 software. The results from three independent experiments were presented as the mean ± S.D. Statistical significance was assessed by two-tailed Student’s *t*-test for two groups. *p <* 0.05 was considered statistically significant.

## 3. Results

### 3.1. Screening of Regulators Affecting the Interaction of UBL3 with α-Syn Using Split Gaussian Luciferase Complementation Assay and RNAi Technology

**Figure 1:**
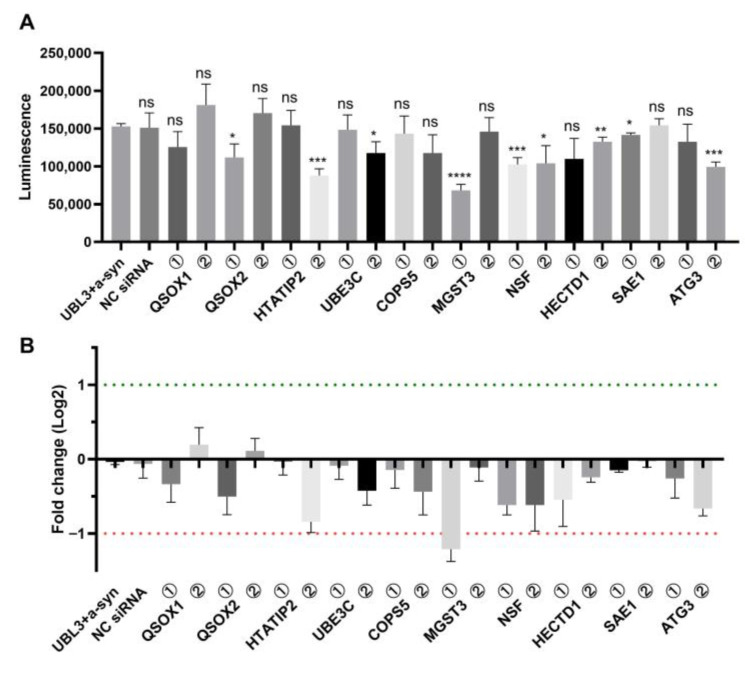
Effect of siRNA on the interaction of UBL3 with α-syn using split-luciferase complementation assay. (**A**) Luminescence of culture medium of HEK293 cells co-transfected with siRNA, NGluc-UBL3, and α-syn-Cgluc. Two siRNAs were available for each candidate protein. The groups were compared with the UBL3 and α-syn co-transfected group, and statistically analyzed. (**B**) Fold change log2 of luminescence. Silencing MGST3 resulted in luminescence fold change log2 < −1. The luminescence ± S.D. in three independent experiments as shown. ns: non-significant; *: *p* < 0.05; **: *p* < 0.01, ***: *p* < 0.001, ****: *p* < 0.0001. NC siRNA: negative-control siRNA, QSOX1: quiescin sulfhydryl oxidase 1, QSOX2: quiescin sulfhydryl oxidase 2, HTATIP2: HIV-1 Tat interactive protein 2, UBE3C: ubiquitin protein ligase E3C, COPS5: COP9 signalosome subunit 5, MGST3: microsomal glutathione S-transferase 3, NSF: N-ethylmaleimide sensitive factor, HECTD1: HECT domain E3 ubiquitin protein ligase 1, SAE1: SUMO1 activating enzyme subunit 1, ATG3: autophagy related 3.

In previous study that discovered UBL3 modification affects the sorting of proteins into sEVs, proteomic analysis was performed to identify proteins that interact with UBL3 in a manner dependent on two c-terminal cysteine residues [2]. From these proteins, we selected 10 candidate proteins based on modification functions involving ubiquitination, glycosylation, acetylation, etc., that may affect the interaction of UBL3 with α-syn. We selected two corresponding siRNAs for each candidate protein (Supplement Table S1). Then, we co-transfected each siRNA with NGluc-UBL3 and α-syn-CGluc into HEK293 cells. After 72 h of incubation, we collected the cell culture medium to detect the luminescence (Figure 1A). Compared to the control group transfected with only NGluc-UBL3 and α-syn-CGluc, the luminescence was significantly downregulated in the group transfected with siRNA silencing QSOX2, HTATIP2, UBE3C, MGST3, NSF, HECTD1, SAE1, and ATG3. After being transformed by fold-change log2 (Figure 1B), we found that silencing MGST3 had the greatest effect on this interaction (fold change log2 < −1). Thus, the QSOX2, HTATIP2, UBE3C, MGST3, NSF, HECTD1, SAE1, and ATG3 are involved in regulating the interaction of UBL3 with α-syn, and MGST3 has the greatest effect.

### 3.2. Confirmation of MGST3 Expression Silencing

**Figure 2:**
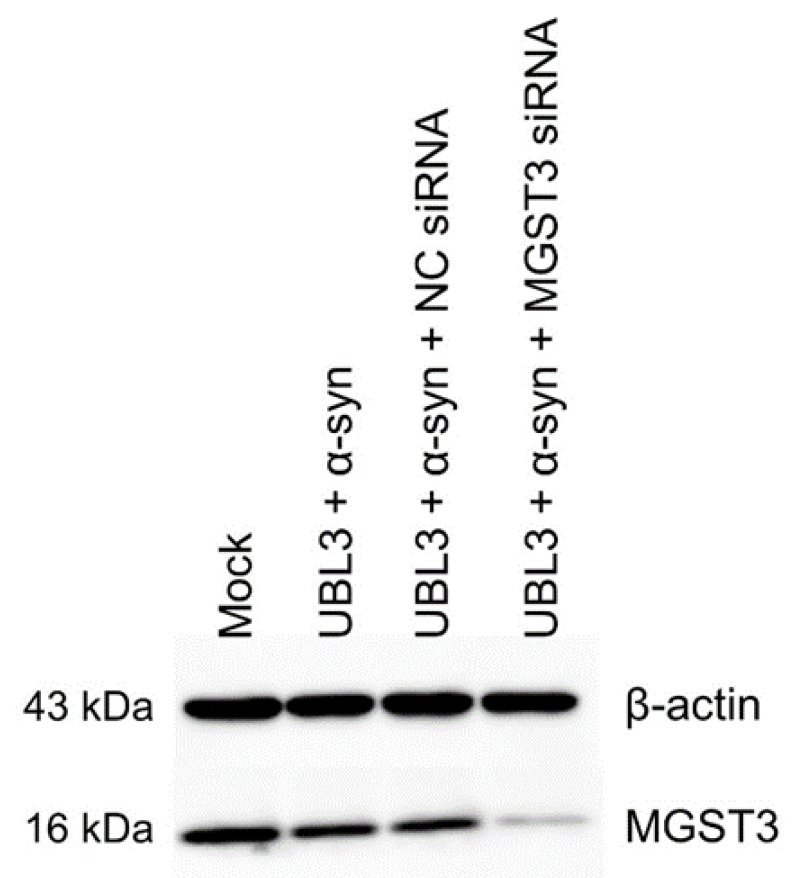
MGST3 was knocked down in HEK293 cells transfected with MGST3 siRNA. NC siRNA: negative-control siRNA.

After quantification of the protein from the cell lysate, we performed western blot analysis to confirm the knockdown of MGST3 expression (Figure 2). We compared the signal of MGST3 in the group transfected with MGST3 siRNA to the control group and found that the signal was attenuated in the MGST3 siRNA-transfected group. This indicated successful knockdown of MGST3 by siRNA in the cells.

### 3.3. Effect of Silencing MGST3 on Co-Localization of UBL3 and α-Syn

**Figure 3:**
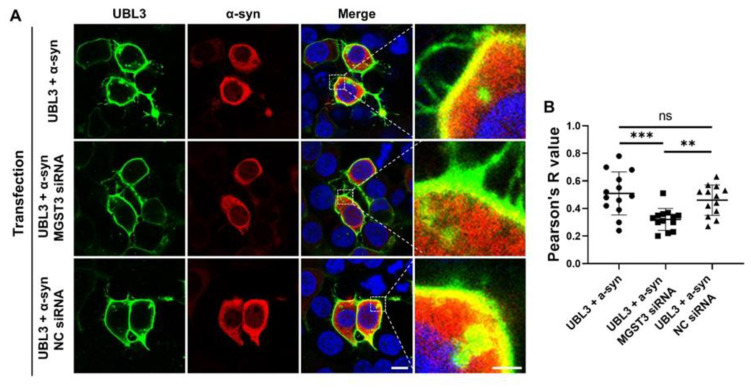
Effect of silencing MGST3 on co-localization of UBL3 and α-syn. (**A**) Representative images of HEK293 cells showing the effect of silencing MGST3 on the co-localization of UBL3 and α-syn. Green represents UBL3, Red represents α-syn, Blue represents cell nucleus and yellow shows the co-localization of UBL3 and α-syn. Scale bars, 10 and 2 μm. (**B**) Quantitative analysis of the effect of silencing MGST3 on co-localization of Flag-UBL3 with MYC-α-syn. (*n* = 13). ns: non-significant; **: *p* < 0.01, ***: *p* < 0.001.

We next examined the co-localization of UBL3 with α-syn in cells, as well as the impact of silencing MGST3 on this co-localization, using immunocytochemical staining. UBL3 was predominantly distributed at the cell membrane and partially in the cytoplasm in a punctate form, and α-syn was mainly diffusely distributed in the cytoplasm (Figure 3A). In all cases, UBL3 appears partially co-localized with α-syn. This co-localization was significantly decreased in the case of MGST3 knockdown (Figure 3). Additionally, the knock down of MGST3 did not have an impact on cell morphology and the intracellular distribution of UBL3 and α-syn.

### 3.4. Effect of Oxidative Stress on the Interaction between UBL3 and α-Syn

MGST3 has mainly glutathione transferase and glutathione peroxidase activities [11,12]. It is known to play a protective role against oxidative stress [13]. Therefore, we verified whether oxidative stress affects the interaction of UBL3 with α-syn.

**Figure 4:**
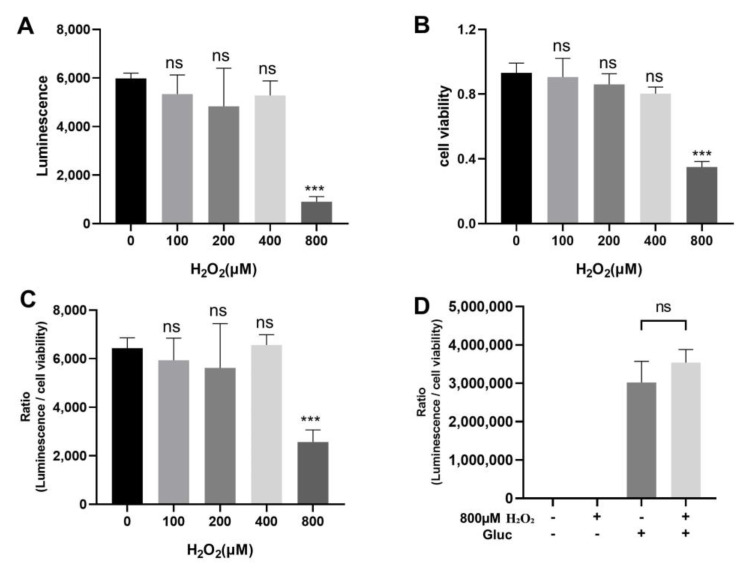
Effect of H_2_O_2_ on the interaction between UBL3 and α-syn. (**A**) Luminescence of culture medium from HEK293 cells co-transfected with NGluc-UBL3 and α-syn-CGluc at different concentrations of H_2_O_2_. (**B**) Effect of different concentrations of H_2_O_2_ on the viability of HEK293 cells co-transfected with NGluc-UBL3 and α-syn-CGluc. (**C**) The ratio of luminescence to cell viability in culture medium from HEK293 cells co-transfected with NGluc-UBL3 and α-syn-CGluc treated with different concentrations of H_2_O_2_. These groups were compared to the group treated with 0 μM H_2_O_2_, and statistically analyzed. (**D**) The ratio of luminescence to cell viability of culture medium from HEK293 cells transfected with Gluc treated with 800 μM H_2_O_2_. The luminescence ± S.D., cell viability ± S.D., and ratio ± S.D. in three independent experiments as shown. ns: non-significant, ***: *p* < 0.001.

An in vitro oxidative stress model has been proposed by using H_2_O_2_ as an inducer when it is added to the cell culture medium [14]. We used cell culture medium containing different concentrations of H_2_O_2_ to culture HEK293 cells transfected with NGluc-UBL3 with α-syn-CGluc. Afterward, the cell culture medium was collected and the luminescence was measured (Figure 4A). Only the 800 μM H_2_O_2_-treated group showed a significant decrease in luminescence compared to the 0 μM H_2_O_2_-treated group. Meanwhile, we observed that the H_2_O_2_ exhibited potent cytotoxicity at a concentration of 800 μM, leading to partial cell death. Therefore, we used the MTT assay to detect cell viability (Figure 4B). Consistent with our observation, the result of MTT assay revealed a significant decrease in cell viability when the H_2_O_2_ concentration was 800 μM. To reduce the effect of cytotoxicity of H_2_O_2_ on the result, we calculated the ratio of luminescence to the cell viability. When the H_2_O_2_ concentration was 800 μM, the ratio decreased significantly compared to the control, while other lower concentrations had no effect (Figure 4C). We treated cells transfected with Gluc using 800 μM H_2_O_2_ and we found that the ratio of luminescence to the cell viability was not different from the group without 800 μM H_2_O_2_ treatment (Figure 4D). The effect of H_2_O_2_ on luciferase activity was excluded. This suggested that a certain level of oxidative stress can downregulate the interaction between UBL3 and α-syn.

### 3.5. Effect of Silencing MGST3 on the Interaction of UBL3 with α-Syn upon Oxidative Stress

**Figure 5:**
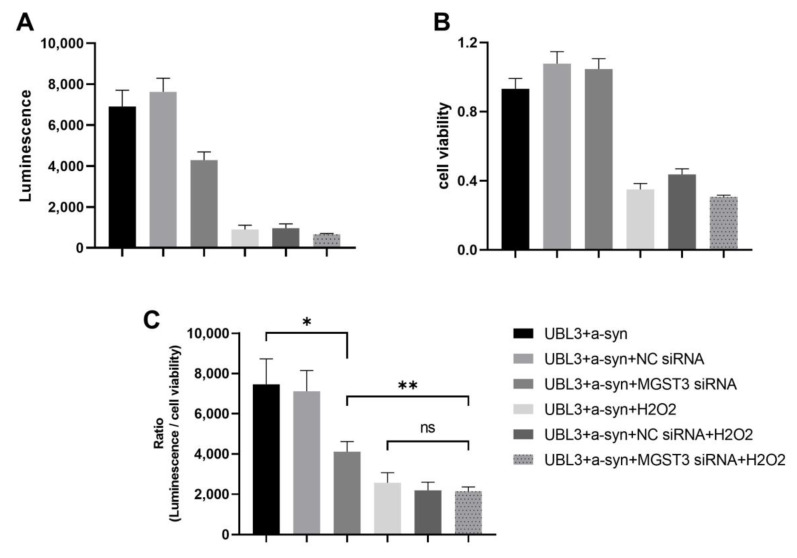
Effect of silencing MGST3 on the interaction between UBL3 and α-syn with 800 μM H_2_O_2_. (**A**) Luminescence of culture medium from HEK293 cells co-transfected NGluc-UBL3 with α-syn-Cgluc. (**B**) Viability of the HEK 293 cells. (**C**) The ratio of luminescence to cell viability of HEK293 cell culture medium co-transfected with Ngluc-UBL3 and α-syn-Cgluc. The luminescence ± S.D., cell viability ± S.D. and ratio ± S.D. in three independent experiments as shown. NC siRNA: negative-control siRNA, ns: non-significant, * *p* < 0.05, ** *p* < 0.01.

Silencing MGST3 could contribute to oxidative stress in cells. It remains unclear whether silencing MGST3 can exacerbate the effects of oxidative stress on UBL3-α-syn interactions or not. To verify this, we treated with H_2_O_2_ at a concentration of 800 μM after performing co-transfection of NGluc-UBL3, α-syn-CGluc, and MGST3 siRNA. Cell culture medium was obtained after incubation for 48 h and assayed for luminescence (Figure 5A). The MTT assay was also used to determine the cell viability and the ratio of luminescence to cell viability was calculated (Figure 5B,C). Although the interaction between UBL3 and α-syn decreased further upon treatment with hydrogen peroxide after silencing MGST3, this effect was not different from the impact of using hydrogen peroxide alone on the interaction between UBL3 and α-syn. This indicated that silencing MGST3 did not affect the downregulation of interaction between UBL3 and α-syn by oxidative stress.

## 4. Discussion

In this study, we found that silencing QSOX2, HTATIP2, UBE3C, MGST3, NSF, HECTD1, SAE1, and ATG3 significantly downregulated the interaction of UBL3 with α-syn, of which silencing MGST3 had the greatest effect.

As an enzyme belonging to the MAPEG (membrane-associated proteins in eicosanoid and glutathione metabolism) family [15], MGST3 catalyzes the conjugation of glutathione to electrophilic compounds, facilitating their removal from cells, and participates in the reduction of H_2_O_2_ and lipid peroxides to their corresponding alcohols [11]. This activity helps to protect cells from oxidative damage and maintain redox homeostasis. It is also commonly used as a biomarker for oxidative stress [16,17]. MGST3 is widely expressed in various human tissues, including the heart, brain, liver, kidney, pancreas, thyroid, skeletal muscle, testis, and ovary [11]. A study using data from a genome-wide association study (GWAS) of brain structure in humans identified a significant positive correlation between MGST3 expression and the size of the hippocampus [18], and the authors suggested that the dysregulation of MGST3 may be associated with neurodegenerative diseases. The aggregation of α-syn is one of the typical pathological features of neurodegenerative diseases, and its pathogenesis involves multiple factors, including neuroinflammation, oxidative stress, protein metabolism disorders, etc. [19,20]. Although MGST3 plays a certain role in these factors, no study has clearly demonstrated that MGST3 affects the aggregation of α-syn. Our finding provides a promising starting point for further investigation of the potential link between MGST3 and α-syn aggregation.

We conducted a screening of proteins that interact with UBL3 and identified MGST3 as a protein that affects the interaction of UBL3 with α-syn. The results of cell staining also provide implicit evidence for this, as the knockdown of MGST3 resulted in a markedly attenuated co-localization of UBL3 with α-syn in the cells. In addition, the overexpressed UBL3 in the cells was predominantly distributed in the cell membrane, which is consistent with previous reports [1,2] that UBL3 is in a membrane-anchored protein. Our results showed that H_2_O_2_ can downregulate the interaction of UBL3 with α-syn. Based on this, we hypothesized that MGST3 can form a complex, MGST3-UBL3-α-syn. The presence of MGST3 can use glutathione to break down H_2_O_2_ in the vicinity of the complex, thereby protecting the UBL3-α-syn interaction from being downregulated by H_2_O_2_. When MGST3 is silenced, cellular oxidative imbalance and increased H_2_O_2_ generation, resulting in downregulation of the interaction of UBL3 with α-syn. Interestingly, under the condition of H_2_O_2_ treatment, we did not observe a further reduction in the interaction of UBL3 with α-syn by MGST3 silencing. We speculate that this may be due to the fact that the intensity of H_2_O_2_ stimulation in this experiment is beyond the range that endogenous MGST3 can effectively regulate. In the future, it would be interesting to investigate whether overexpression of MGST3 can prevent the downregulation of the UBL3-α-syn interaction by H_2_O_2_.

It is still unclear how H_2_O_2_ alters the interaction of UBL3 with α-syn based on the information currently available. The C-terminal cysteine residues of UBL3 are essential for the post-translational modification of the target protein with UBL3 [2], but that modification seemed not to occur to the interaction of UBL3 with α-syn [10]. It is possible that H_2_O_2_ may affect the intracellular interaction of UBL3 with α-syn by altering the covalent modification of UBL3 with other unknown factors mediated by the C-terminal cysteine residues. Notably, in our previous study it was found that 1-methyl-4-phenylpyridinium (MPP+), which can cause mitochondrial dysfunction and lead to reactive oxygen species production, can upregulate the interaction of UBL3 with α-syn [10]. This is inconsistent with the finding that direct treatment of cells with H_2_O_2_ resulted in a decrease in the interaction of UBL3 with α-syn. We cannot exclude the possibility that the MPP+ induced rise in this interaction is due to the fact that MPP+ can increase the expression of α-syn [21], an effect that outweighs the effect of the oxidative stress induced by MPP+ on this interaction. However, we need more experiments to investigate this.

Oxidative stress caused by the excessive production of reactive oxygen species such as H_2_O_2_, hydroxyl radicals, and superoxide from cellular metabolic processes leads to cell damage. Growing evidence indicates that oxidative stress is one of the crucial factors involved in the pathogenesis of neurodegenerative diseases [22,23]. For example, the brain of PD patients displays decreased mitochondrial function and excessive production of ROS, including the depletion of endogenous antioxidants and oxidative damage to cellular macromolecules such as proteins, lipids, and nucleic acids [24,25]. It has been found that the production of ROS can trigger the formation of Lewy bodies [26]. In addition, increased levels of cholesterol metabolites of ROS have been reported in the cerebral cortex of patients with DLB, and it has also been shown that increased levels of such metabolites accelerate the aggregation of α-syn [27]. Our finding that the interaction between UBL3 and α-syn was downregulated by oxidative stress prompted inquiries into its potential contribution to neurodegenerative processes. The downregulation of UBL3’s interaction with α-syn by oxidative stress promotes the release of α-syn from the UBL3-α-syn complex, disrupting the transfer of α-syn to the extracellular compartment by UBL3. This interferes with the potential role of UBL3 in preventing α-syn aggregation within the cell, leading to the accumulation of α-syn and the formation of aggregates. Further investigation is needed to fully understand the underlying mechanisms involved in this process, and to determine how these factors may contribute to the development of neurodegenerative diseases.

In this study, some limitations remain. Although we found that silencing of MGST3 downregulated UBL3 interaction with α-syn by split Gluc assay and cellular immunofluorescence staining, molecular techniques including immunoprecipitation, real-time PCR, and in vivo experiments in mice need to be implemented to further validate our results. Meanwhile, it is still necessary to investigate whether proteins with similar function and structure as MGST3 also have the ability to affect the UBL3–α-syn interaction. Moreover, the specific mechanisms and subcellular sites where MGST3 and H_2_O_2_ are involved in influencing this interaction remain unclear. All of this needs to be studied and explored in more depth in future experiments.

## 5. Conclusions

In conclusion, our findings suggest that MGST3 plays a role in regulating the interaction between UBL3 and α-syn.

## Data Availability

All relevant data were reported within the article. Further supporting data will be provided upon a written request addressed to the corresponding author.

## Supplementary Materials

The following supporting information can be downloaded at: https://www.mdpi.com/2227-9059/11/9/2491/s1, Table S1: The list of siRNAs used in the current study.
