# Supplementary material for: UBL3 Interaction with α-Synuclein Is Downregulated by Silencing MGST3
Source: Biomedicines. 2023 Sep 8;11(9):2491. doi: 10.3390/biomedicines11092491 (PMC10648775; doi:10.3390/biomedicines11092491)
Supplement: Supplementary file 1 [file biomedicines-11-02491-s001.zip › Supplementary File.pdf]

Supplemental Table S1

The list of siRNAs used in the current study

**siRNA targeting**	**siRNA ID***	**Sense 5′- 3′**	**Antisense 5′- 3′**
QSOX1	s224485	CCU GCA CUC CGU GAA UGA Att	UUC AUU CAC GGA GUG CAG Gaa
	s11505	GGA UUC UUU GCG AGA AAU Att	UAU UUC UCG CAA AGA AUC Cat
QSOX2	s46761	GAA UAU UCC UUA CUA AUC Att	UGA UUA GUA AGG AAU AUU Cca
	s46763	GAG GUG AUC UUA GAC CUG Att	UCA GGU CUA AGA UCA CCU Ccc
HTATIP2	s230128	CGA CGA GGA AGC UUA UAA Att	UUU AUA AGC UUC CUC GUC Gaa
	s230129	AGA GUG CUC UUA AAG GAA Att	UUU CCU UUA AGA GCA CUC Ugc
UBE3C	s18661	CAG GGU CUA UGG UAC CGU Utt	AAC GGU ACC AUA GAC CCU Gtg
	s18660	GAG ACA CUU UUG CGA AGU Att	UAC UUC GCA AAA GUG UCU Cgt
COPS5	s21627	GCA AUC GGG UGG UAU CAU Att	UAU GAU ACC ACC CGA UUG Cat
	s21629	GGA UUG AUG UUA GUA CUC Att	UGA GUA CUA ACA UCA AUC Cca
MGST3	s8762	AGA ACA CGU UGG AAG UGU Att	UAC ACU UCC AAC GUG UUC Ugg
	s8764	GAC GAG UUC UUU AUG CUU Att	UAA GCA UAA AGA ACU CGU Cca
NSF	s181	GUU ACA UUA UGA ACG GUA Utt	AUA CCG UUC AUA AUG UAA Ctt
	s182	GGC AGA CUU UCU ACA UGC Att	UGC AUG UAG AAA GUC UGC Ctt
HECTD1	s24575	GGC UCG AGA UUU AUA CGA Utt	AUC GUA UAA AUC UCG AGC Cat
	s24576	CAG UUG CGU UAA UUC GAA Att	UUU CGA AUU AAC GCA ACU Gct
SAE1	s19551	GUU CCG UAC AGA UAA AGG Att	UCC UUU AUC UGU ACG GAA Ctt
	s19552	GCU GGA UCA CGA ACA GGU Att	UAC CUG UUC GUG AUC CAG Cat
ATG3	s34731	GAA UAA CGG AAG CCG UUA Att	UUA ACG GCU UCC GUU AUU Cct
	s34733	GCG GAU GGG UAG AUA CAU Att	UAU GUA UCU ACC CAU CCG Cca

*Silencer Select (Ambion, Life Technologies)
